# Investigating heartbeat-related in-plane motion and stress levels induced at the aortic root

**DOI:** 10.1186/s12938-019-0632-7

**Published:** 2019-02-26

**Authors:** Wei Wei, Morgane Evin, Stanislas Rapacchi, Frank Kober, Monique Bernard, Alexis Jacquier, Cyril J. F. Kahn, Michel Behr

**Affiliations:** 1 Laboratoire de Biomécanique Appliquée, Aix-Marseille Université, IFSTTAR, LBA, UMR T24, 51 Bd. P. Dramard, 13015 Marseille, France; 2 Aix-Marseille Université, CNRS, CRMBM, UMR 7339, Marseille, France

**Keywords:** Aortic root motion, Magnetic resonance imagining, Aortic stress, Finite element, Fluid–structure interaction

## Abstract

**Background:**

The axial motion of aortic root (AR) due to ventricular traction was previously suggested to contribute to ascending aorta (AA) dissection by increasing its longitudinal stress, but AR in-plane motion effects on stresses have never been studied. The objective is to investigate the contribution of AR in-plane motion to AA stress levels.

**Methods:**

The AR in-plane motion was assessed on magnetic resonance imagining data from 25 healthy volunteers as the movement of the AA section centroid. The measured movement was prescribed to the proximal AA end of an aortic finite element model to investigate its influences on aortic stresses. The finite element model was developed from a patient-specific geometry using LS-DYNA solver and validated against the aortic distensibility. Fluid–structure interaction (FSI) approach was also used to simulate blood hydrodynamic effects on aortic dilation and stresses.

**Results:**

The AR in-plane motion was 5.5 ± 1.7 mm with the components of 3.1 ± 1.5 mm along the direction of proximal descending aorta (PDA) to AA centroid and 3.0 ± 1.3 mm perpendicularly under the PDA reference system. The AR axial motion elevated the longitudinal stress of proximal AA by 40% while the corresponding increase due to in-plane motion was always below 5%. The stresses at proximal AA resulted approximately 7% less in FSI simulation with blood flow.

**Conclusions:**

The AR in-plane motion was comparable with the magnitude of axial motion. Neither axial nor in-plane motion could directly lead to AA dissection. It is necessary to consider the heterogeneous pressures related to blood hydrodynamics when studying aortic wall stress levels.

**Electronic supplementary material:**

The online version of this article (10.1186/s12938-019-0632-7) contains supplementary material, which is available to authorized users.

## Background

Aortic dissection is rare but a potentially life-threatening illness. Apart from hypertension and aortic dilation [1], the aortic root (AR) motion has also been proposed to be another factor leading to dissection [2, 3]. During the cardiac cycle, the aortic annulus is towed due to ventricular traction in systole and is relaxed in diastole. The traction force induces a spatial movement of the aortic annulus and is transmitted to the ascending aorta (AA). The AR motion has been proved to alter in parallel with such cardiovascular pathologies as left ventricular hypokinesis and aortic insufficiency [2]. Since supra-aortic vessels were relatively constrained compared to AA, different AR motions would bring about different levels of aortic wall stress, which was proposed as a risk prediction index for aortic dissection [4] and aortic aneurysm [5].

A mean value of 8.9 mm (range 6.4–11.3 mm) for aortic motion was observed along the lumen longitudinal direction with cine-magnetic resonance imaging (MRI) studies in healthy subjects [6]. The aortic downward displacement was also reported to range between 0 and 49% of the sino-tubular junction diameter in patients with coronary artery diseases [2]. In contrast, the mean in-plane (perpendicular to the lumen) displacement of AA was respectively reported as 5.2 ± 1.7 mm for patients with chronic aortic dissection type B [7] and 6.7 ± 1.8 mm for the young healthy volunteers [8]. However, the component displacements in the anterior–posterior or the lateral direction were not mentioned in both studies.

Aortic finite element (FE) models were previously used to evaluate the AR downward [2, 3, 9] and twisted [2, 3] motion effects on proximal AA stress levels. Studying the influences of AR in-plane movement is however limited. A lack of model validation against physiological data might also undermine the accuracy of aortic stress. Moreover, an uniformly distributed loading was assumed on the aortic wall in these previous studies while the simulation fidelity could benefit from considering the inhomogeneous pressure ambient due to blood flow [10–12].

Therefore, the aim of our study was threefold. Firstly, in order to add additional knowledge to AR physiological motion, the in-plane components of heartbeat-related AR displacement will be evaluated in healthy volunteers with MRI data. Secondly, the fluid–structure interaction (FSI) will be simulated between the aortic wall and blood to assess the fluid dynamic effects on aortic stress levels. Finally, to determine the in-plane motion effects on AA dissection risks, the AA stress levels will be studied under different AR motions with a validated FE model.

## Materials and methods

### Volunteers

The study was approved by the local ethics committee (CPP Sud Méditerranée I, Marseille, ID RCB 2012-A01093-40) and the written informed consent was granted by each volunteer. Twenty-five volunteers (15 men and 10 women, mean age 30.4 ± 9.7 years, mean height 175.8 ± 7.6 cm, mean weight 65.8 ± 13.0 kg) were recruited into this evaluation and the candidates had to match the following criteria: no history of cardiovascular disease, hypertension, diabetes or hypercholesterolemia.

### Image acquisition and evaluation

**Fig. 1:**
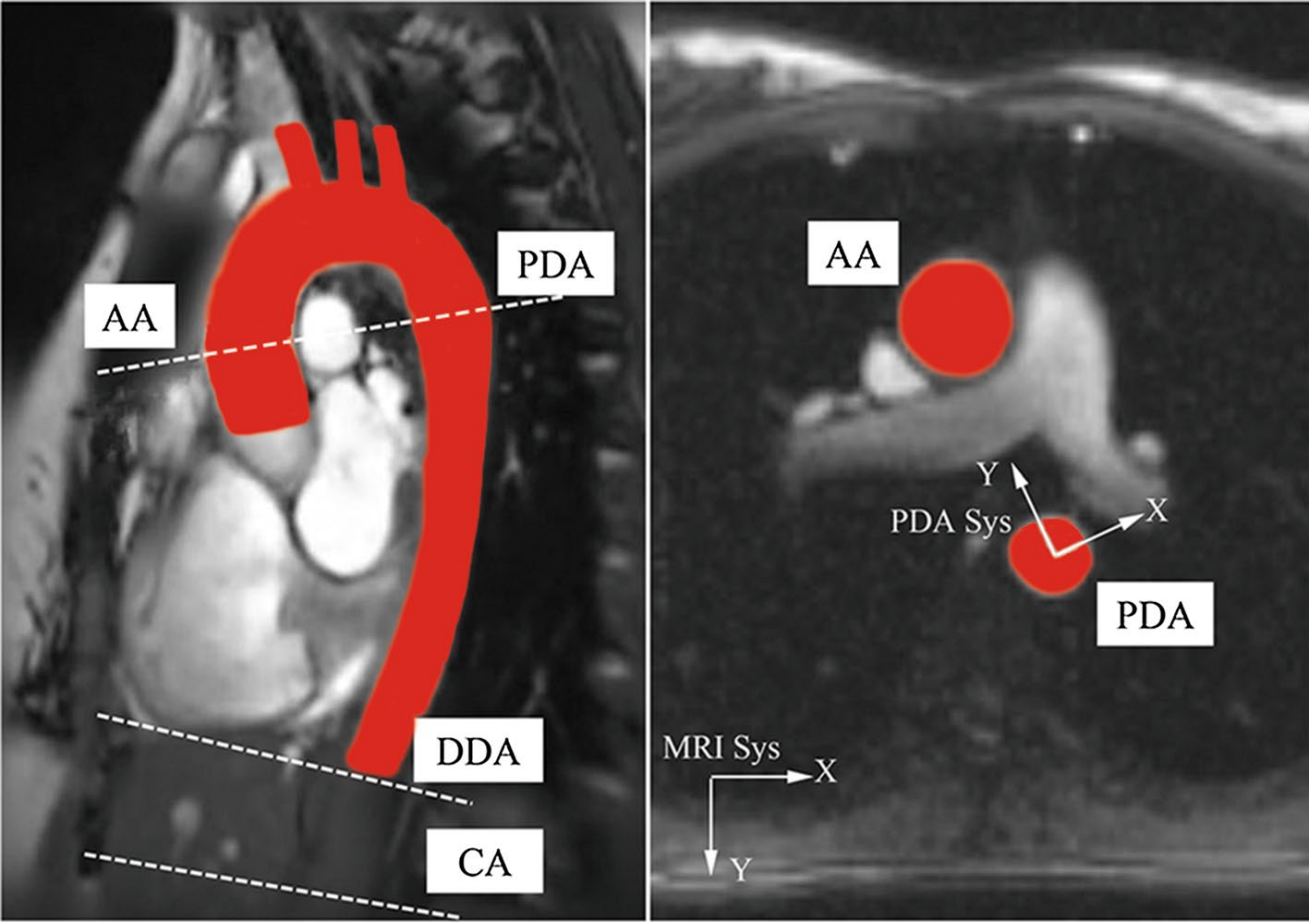
Oblique sagittal and AA-perpendicular MRI images. The region in red colour corresponds to the aorta. Left: oblique sagittal MRI image showing the locations of different aortic sections. Right: the MRI image perpendicular to the AA for measuring its in-plane motion. MRI Sys and PDA Sys are the abbreviations for MRI reference system and PDA system respectively

The image acquisition was performed for all the subjects during a breath-hold with a 1.5 T MRI scanner (Avanto VB17, Siemens, Erlagen, Germany) under a protocol as previously described in [13]. A stack of segmented steady-state free precession (SSFP) bright blood images were acquired in axial and oblique sagittal planes (Fig. 1) to assess the aortic slice segmentation. SSFP cine images were subsequently obtained at three different levels [AA together with the proximal descending aorta (PDA) at the level of pulmonary trunk, the distal descending aorta 3 cm above the diaphragm (DDA) and above the coeliac trunk (CA)] perpendicular to the aortic lumen (Fig. 1) with the following parameters: TR = 21.7 ms to 24.7 ms, TE = 1.36 ms to 1.55 ms, α = 65°, recFOV = 210 mm × 263 mm to 280 mm × 340 mm, slice thickness = 7 mm, pixel size = 1.26 mm × 1.26 mm to 1.68 mm × 1.68 mm. It is worth noting that only the images at AA section were used to evaluate the AR in-plane motion.

Dynamic datasets were loaded into a semi-automatic tool, Argus (Siemens, Erlangen, Germany), in which the region of interest (ROI) was created and the aortic lumen boundary was detected based on the intensity gradient. After being manually adjusted, the ROI was propagated and adapted for each phase of the cardiac cycle. The Cartesian coordinates of the points on the aortic contour could be provided by this software application. The ROI geometric centroid was obtained by averaging the coordinates of the aortic contour. The AA in-plane displacement was defined as the distance between the centroid at the ending of diastole (initial) and the centroid on the analysed image. The mean value and the standard deviation among all the subjects were then calculated from the maximum in-plane displacement of each time series. A PDA system was constructed with its origin at the PDA centroid at the ending of diastole, with the positive Y direction from the origin to AA centroid and with positive X normal to Y axis pointing to the left (Fig. 1).

### Reconstruction of the aorta

The aortic lumen was detected from end-diastolic 2-dimensional stack of SSFP images of a randomly-selected volunteer (25 years, male) using the in-plane region-growing method on Mimics software (Marterialise, Louvain, Belgium). The sinus of valsalva was not reconstructed since the AR was not the focus of the aortic wall stress analysis. As the 3-dimensional surface evolution was run through the stack of segmented contour, the aortic geometry was then extracted, smoothed and exported with stereolithography version for later processing. Since the resolution of the image acquisition was not high enough to detect the thickness of the aortic wall, the geometry reconstructed here was assumed to be the inner wall of the aorta.

### FE modelling and material properties

LS-Prepost (LSTC, Livermore, CA, USA) was used to discretize the aortic inner wall with 4-node shell elements, which were subsequently offset outward with a uniform thickness of 1.6 mm to generate the hexahedral elements for the aortic wall. The assumed uniform aortic thickness was compatible with the reported ranges in literature [14] and was also commonly performed as in previous works [2, 9]. The shell elements of the aortic inner wall were only used to generate the aortic wall brick elements only and not for the following simulations.

In order to determine the aortic model size, a mesh convergence analysis was performed with a pure structural simulation. A pressure of 80 mmHg was imposed on aortic exterior walls of three models which were respectively discretized with 3.0e+4, 1.0e+5 and 3.0e+5 brick elements (resulting in 4.5e+4, 1.3e+5 and 3.8e+5 nodes). The aortic wall stresses were compared among these models. The model with 1.0e+5 elements and 1.3e+5 nodes was found converged (detailed in Additional file 1: Appendix S1) and was thus chosen for the following analysis.

**Fig. 2:**
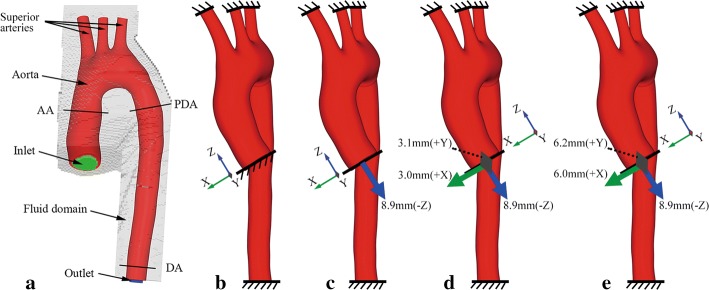
Aortic FE model and boundary conditions. Aortic FE model for structural and FSI simulation (**a**). Boundary conditions for SA-Pre (**b**), SA-Down (**c**), SA-XY (**d**) and SA-2XY (**e**). AA, PDA and DDA correspond to the sections of distensibility assessment

Since the initial aortic geometry was reconstructed at end-diastole, the intra-aortic pressure was about 80 mmHg [15] instead of a zero-pressure condition. The aorta stress-free configuration was achieved by extracting the resulting deformed mesh from mesh convergence simulation in which a pressure of 80 mmHg was imposed on the aortic external wall to offset the end-diastole pressure, as previously done in [16]. In order to simulate the blood flow and study the hydrodynamic effects, a fluid domain (Fig. 2) was constructed to immerse the stress-free aorta. The fluid part was discretized with 250,000 hexahedral Eulerian elements (2.6e+5 nodes). This size was also decided after a mesh convergence analysis against the section-averaged blood velocity with a 1% threshold.

The aortic wall was assumed to be transversely isotropic and incompressible hyper-elastic material, the material equation and parameters (*C*_1_ = 191 kPa, *C*_2_ = 0.451 and *C*_3_ = *C*_4_ = 0.184) of which came from a previous numerical study [17]. The fluid Eulerian mesh was subdivided in two domains and defined as multi-material: the first domain was blood initiated inside the aorta; the second domain was the fluid part outside of the aortic wall and defined as vacuum. The two fluid domains always updated as the FSI interface (i.e. aortic wall) moved or deformed, maintaining the blood inside the aorta and vacuum outside. For simplification, the blood was assumed as Newtonian fluid [16] with a density of 1050 kg/m^3^, a dynamic viscosity set to 4.5e−3 Pa s and a bulk modulus of 2.5 GPa [16]. The relationship between blood pressure and volume (density) was described by a linear equation of state (detailed in Additional file 2: Appendix S2).

### Aortic FE model validation

In order to validate the bio-fidelity of the aortic FE model, a structural simulation was performed on the stress-free configuration with three cycles of physiological time-dependent pressure [18] distributed on the inner surface of aortic wall. The aortic diameters of AA, PDA and DDA (see Fig. 2a) were recorded during the simulation and those during the third cycle were used to assess the aortic distensibility. The distensibility was calculated with the Eq. (1) [19]:

1 $$ {\text{Distensibility }}\;\left( {10^{ - 3} \;{\text{mmHg}}^{ - 1} } \right) = \frac{{A_{max} - A_{min} }}{{A_{min} }} \times P_{pulse} = \left[ {\left( {\frac{{D_{max} }}{{D_{min} }}} \right)^{2} - 1} \right] \times P_{pulse} $$

where *A*_*max*_ and *A*_*min*_ represent the maximal and minimal aortic cross-sectional areas during the cycle, *P*_*pulse*_ is the pulse pressure and *D*_*max*_ and *D*_*min*_ are respectively the maximal and minimal aortic diameters.

### Boundary and loading conditions

Four simulations were performed for structural analysis and two for FSI simulations, all of which were conducted on the stress-free configuration. The distal ends of the superior arteries and the descending aorta were constrained during all the simulations. The proximal end of AA was fully constrained only for the simulations without AR motion prescribed (Fig. 2b–e). All of the simulations (mesh convergence, FSI and structural analysis) were performed with the solver LS-DYNA 971 R7.1.1 (LSTC, Livermore, CA, USA) on an Intel Xeon (2.57 GHz) workstation with 40 processors.

For FSI analysis, the solver LS-DYNA computes fluid and structure physics separately, and then couples the two physics until equilibrium is reached [20]. The interaction between fluid and structure domain was modelled by activating the penalty coupling algorithm in LS-DYNA, in which elastic forces were computed against the structure-fluid penetration and imposed on the structural elements. The inlet and outlet (Fig. 2a) fluid parts were applied with a constant pressure of 120 mmHg for a static analysis (hereafter referred as *FSI*-*Sta*). Another hydrodynamic simulation was conducted by pressurizing the inlet with 120 mmHg and prescribing an outflow of 300 mL/s at the outlet (referred as *FSI*-*Flow*). The reason why a constant pressure and flow rate rather than a pulsatile blood flow was chosen to apply in FSI analysis was to better compare the aortic stress levels in FSI and in structural analysis.

For the structural analysis, a Cartesian coordinate system was constructed to prescribe the AA motion (Fig. 2) according to the local PDA system for AA in-plane measurement (Fig. 1). A pressure of 120 mmHg inside the aortic wall was the only loading for the control model (referred as *SA*-*Pre* and see Fig. 2b). Besides 120 mmHg pressurized inside the aortic wall, a displacement of 8.9 mm along −Z was applied to AA proximal end to simulate AR downward traction (referred as *SA*-*Down* and see Fig. 2c). The corresponding displacements (3.0 mm—X, 3.1 mm—Y) obtained from the cine MRI analysis were further imposed on the AA proximal end to evaluate the effects of AA in-plane displacement (referred as *SA*-*XY* and see Fig. 2d). Finally, considering the hypothesis of AA in-plane displacement equal with AR motion, the AA proximal end was prescribed with twice magnitudes (6.0 mm—X, 6.2 mm—Y) of the in-plane displacement in another simulation (referred as *SA*-*2XY* and see Fig. 2e) to aggressively estimate its influences.

## Results

### AA in-plane motion

**Fig. 3:**
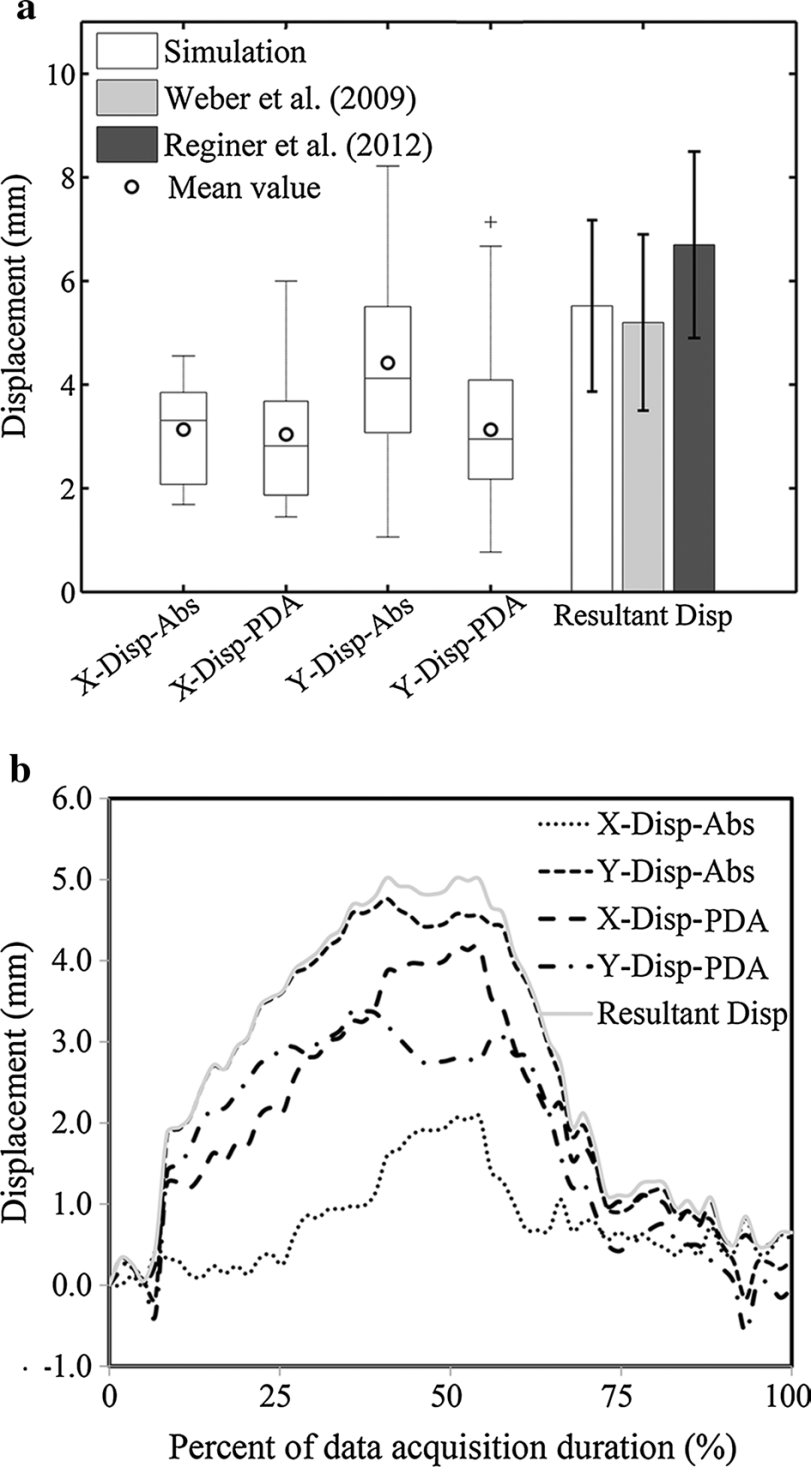
In-plane motion of AA section. AA maximal in-plane motion in absolute value averaging among the volunteers (**a**); time-history of in-plane resultant and component displacements of a volunteer (25 years, male) (**b**). X-Disp-Abs and X-Disp-PDA: X component motion under MRI and PDA reference system; Y-Disp-Abs and Y-Disp-PDA: Y component motion under MRI and PDA reference system; Resultant Disp: in-plane resultant displacement

Mean value (± standard deviation) of AA in-plane maximal resultant displacement was 5.5 ± 1.7 mm with X and Y components respectively: 3.1 ± 0.9 mm and − 4.4 ± 1.7 mm (Fig. 3a) under the MRI reference coordinate system. When measured in local PDA system, the X and Y components were correspondingly 3.0 ± 1.3 mm and 3.1 ± 1.5 mm (Fig. 3a). AA in-plane motion had two phases: the displacement increased and oriented left-anteriorly during systole and then regressed to its origin in diastole (Fig. 3b).

### Aortic distensibility

**Fig. 4:**
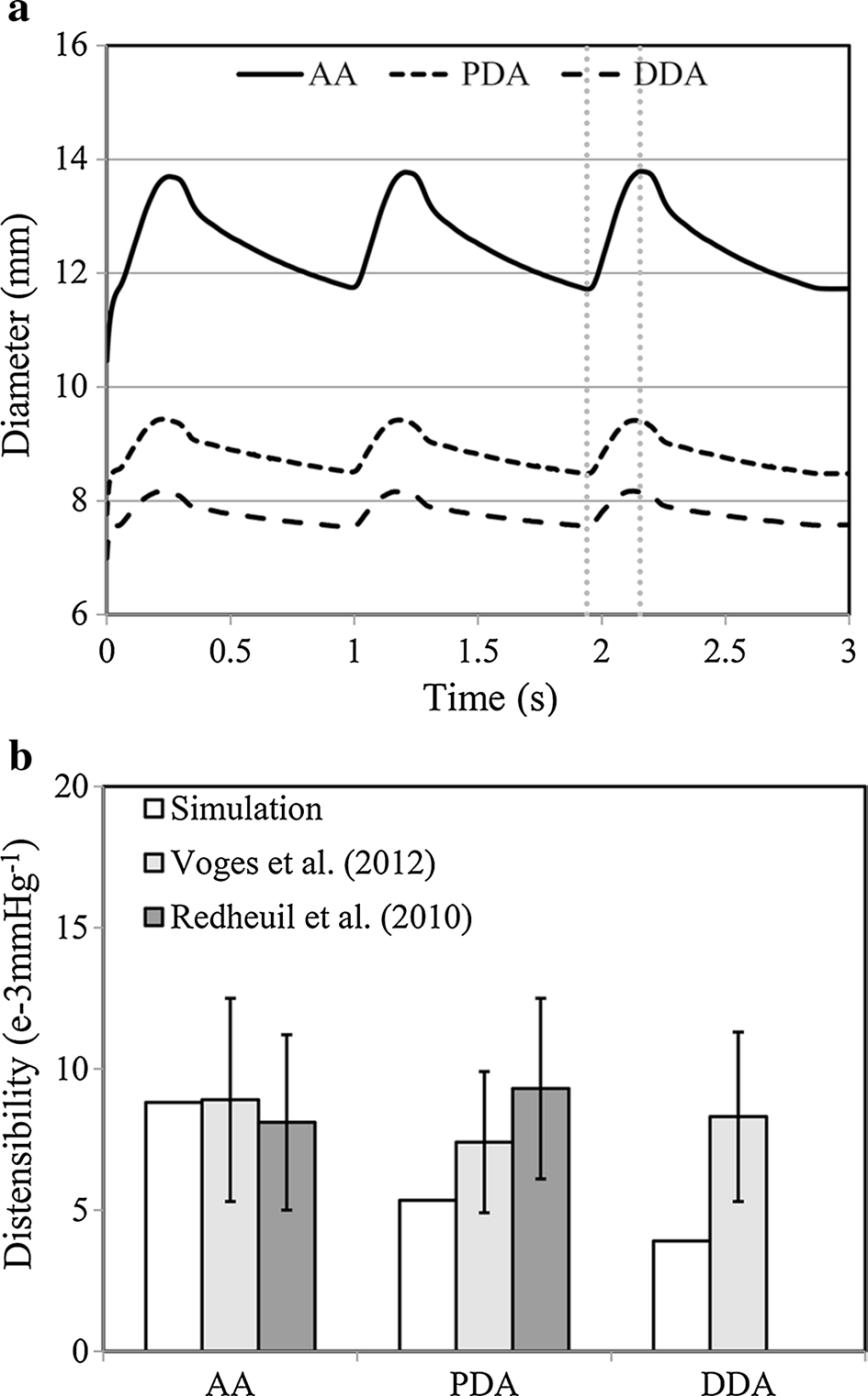
Aortic diameters and distensibility for AA, PDA and DDA. Diameter time-history under the three-cycle pressure loading (**a**); aortic distensibility in simulation comparing with the literature data (**b**). The vertical dotted lines indicate the moments when the diameters were recorded for distensibility analysis

During numerical validation, the diameters of AA, PDA and DDA were 11.7 mm, 8.5 mm and 7.5 mm respectively at the beginning of systole and 13.8 mm, 9.4 mm and 8.2 mm at the ending of systole (Fig. 4a). The distensibility was correspondingly 8.8e−3 mmHg^−1^, 5.3e−3 mmHg^−1^ and 3.9e−3 mmHg^−1^ for AA, PDA and DDA (Fig. 4b).

### FSI and structural analysis

The distributions of von Mises, circumferential and longitudinal stress (see Additional file 2: Appendix S2) were similar among the control model (*SA*-*Pre*) and the FSI simulations (*FSI*-*Sta* and *FSI*-*Flow*). The peak von Mises and circumferential stress occurred at the interior curvature of aortic arch with the corresponding values of 0.24 MPa and 0.48 MPa for *FSI*-*Sta* and 0.22 MPa and 0.47 MPa for *FSI*-*Flow*. The peak longitudinal stress was 0.43 MPa for static *FSI*-*Sta* and 0.42 MPa for *FSI*-*Flow*, both located at the superior artery intersection. In Table 1 were displayed the aortic luminal volumes, AA, PDA and DDA sectional diameters in control and FSI simulations. The averaged stress levels, which were evaluated at the proximal AA section 2 cm above the AR, were also displayed in Table 1.

**Table 1:** Aortic volumes, diameters of different sections and the averaged stress at the proximal AA section 2 cm above the AR in control model and FSI simulations

	Volume (mL)	Diameter (mm)			Stress (e−2 MPa)		
		AA	PDA	DDA	VonM	Circum	Long
SA-Pre	163.1	29.9	19.8	16.9	9.3	14.4	6.0
FSI-Sta	163.0	29.9	19.8	16.9	9.3	14.3	6.0
FSI-Flow	158.3	29.6	19.4	16.4	8.7	13.3	5.6
Diff-Sta (%)	− 0.1	0.0	0.0	0.0	0.0	0.0	0.0
Diff-Flow (%)	− 3.0	− 1.1	− 2.0	− 2.9	− 6.6	− 7.5	− 6.4

The Diff-Sta represented the result difference (in percent) between SA-Pre and FSI-Sta; Diff-Flow represented the result difference (in percent) between SA-Pre and FSI-Flow

*VonM* von Mises, *circum and long* circumferential and longitudinal stresses

**Fig. 5:**
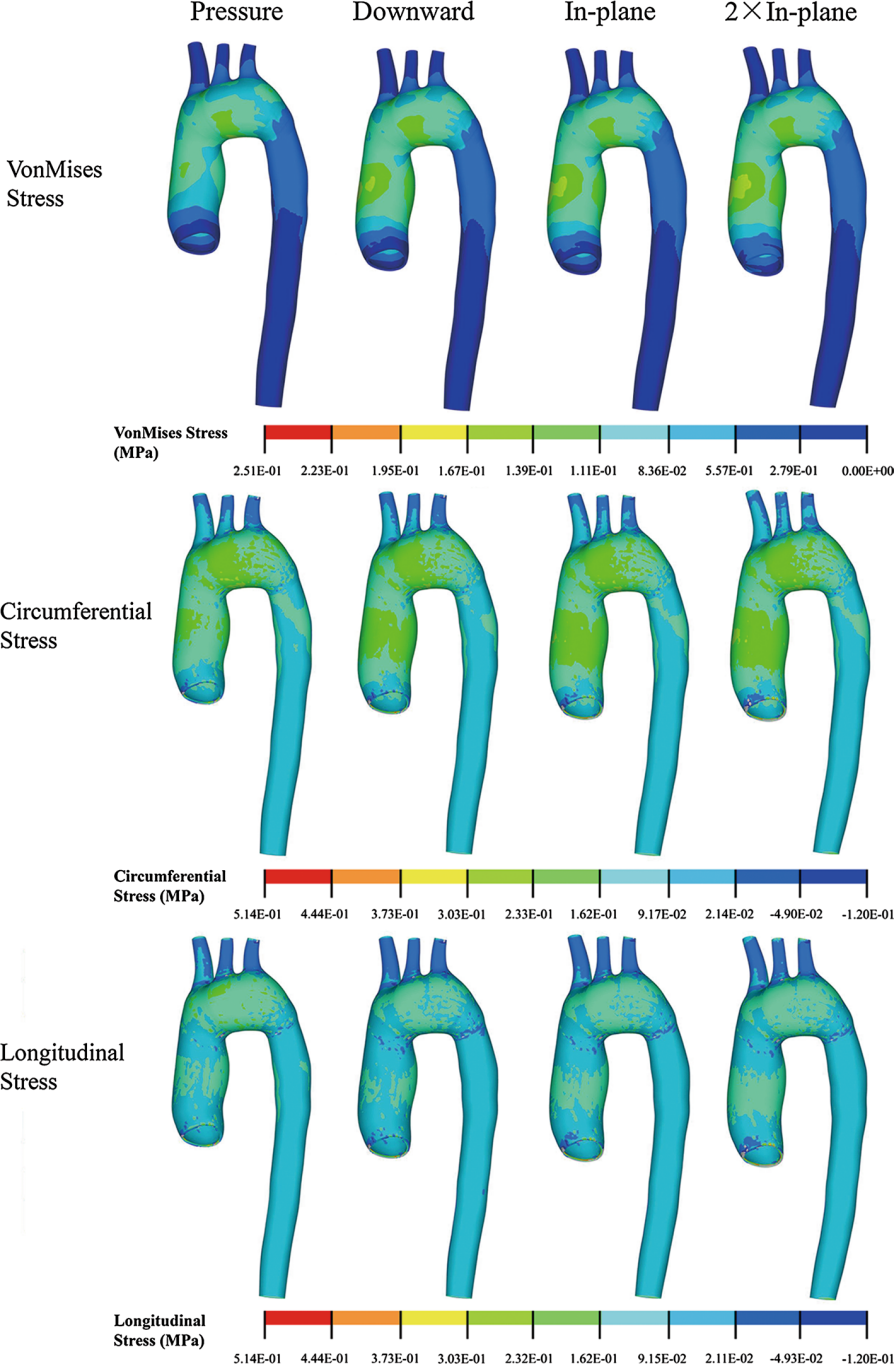
von Mises, circumferential and longitudinal stress distribution for structural simulations with different AA motions

The von Mises and circumferential stress contours were similar among all the structural simulations (Fig. 5), with the corresponding peak values approximately 0.25 MPa and 0.50 MPa located at the interior curvature of aortic arch distal to AA. The longitudinal stress distributions (Fig. 5) were also similar under different loadings with the superior artery intersection region always subjected to a peak stress of 0.43–0.51 MPa. The peak circumferential stretch ratio of aortic wall (not shown) was 1.48 for all the structural simulations. The peak longitudinal stretch ratio (not shown) was 1.37 for *SA*-*Pre* and 1.41 for the other 3 structural simulations with AR motions (*SA*-*Down*, *SA*-*XY* and *SA*-*2XY*). The averaged stress levels at proximal AA section 2 cm above AR were displayed in Table 2 for the structural simulations.

**Table 2:** Averaged von Mises, circumferential and longitudinal stress for proximal AA 2 cm above the AR in structural simulations

Stress (MPa)	Loadings			
	SA-Pre	SA-Down	SA-XY	SA-2XY
von Mises	0.093	0.128	0.131	0.134
Circumferential	0.144	0.191	0.195	0.199
Longitudinal	0.060	0.084	0.086	0.083

## Discussion

### Ascending aortic in-plane motion assessment

In this study, the AA in-plane motion was analysed under MRI and PDA reference system. Although seeming to be more explicit under MRI system, the in-plane displacement under PDA system might be more meaningful with the specific anatomic reference at PDA. The component motions under PDA system were mostly alike. The Y component mean value was 42% higher than the X component under MRI system resulting in a left-anteriorly oriented motion. Similarly, the AA in-plane motion was reported to be left-anterior in 58% cases and to be anterior in 43% [21]. Moreover, the Y component was found to be nearly twice of the X component [21]. The resultant in-plane displacement in our study was also consistent with the published values 5.2 ± 1.7 mm [7] and 6.7 ± 1.8 mm [8], all of which were comparable with the magnitude of downward motion (8.9 ± 1.8 mm) [6]. This also justified the necessity to study the in-plane motion effects on aortic stresses.

Weber et al. [21] indeed studied the aortic 4-dimensional displacement with computed tomography angiography, but the final temporal resolution as well as the temporal reconstruction methods lacked of description. In CT scan, the normal range of temporal resolution was around 83–125 ms according to another research [22]. In contrast, the time resolution of our MRI dataset was about 15 ms, enabling to capture a more detailed in-plane motion during a 300 ms-long systole. Admittedly, the influences of AA through-plane displacement on its in-plane measurement had to be ignored due to the limited computational capabilities to analyse the 4-dimensional dataset. Besides, the AR in-plane displacement also had to be assumed equal to the AA motion with the current data accessible. Despite the hypothesis above, our AA in-plane motion analysis could still add to the knowledge of aortic 3-dimensional motion related to the cardiac pulsatility.

### Model assessment against aortic distensibility

Before studying the AA in-plane motion effects, the distensibility of the model was analyzed to evaluate its bio-fidelity. Although lower than the published mean values [19, 23], the distensibility for AA and PDA was within their standard deviations. The DDA of the model seemed to be less compliant than reported [19]. The fixed boundary at distal DDA could have limited the radial inflation of DDA. The aorta was assumed to be of 1.6 mm uniform thickness in this study for modelling convenience and the difficulties to detect the aortic thickness with our available MRI data. However, the descending aorta has been suggested about 15% thinner than AA [24]. The relative thicker descending aorta in the simulation was speculated to induce its lower distensibility. Still, the reproduced circumferential stretch ratios at peak systole of AA, PDA and DDA were respectively 1.32, 1.21 and 1.17. These values coincided with the published ranges 1.08–1.47 (median value 1.26) obtained with the same pressure level inflation tests [14]. Therefore, to some extent, this aortic FE model was still believed to reflect the realistic aortic compliance under physiological conditions.

### Necessity to consider fluid–structure interactions

Fluid dynamic effects on aortic responses were also analysed by comparing the results of the control model and the FSI simulation with or without a constant blood flow. The differences against the control model were always no more than 0.1% in terms of aortic luminal volumes, diameters or stresses when a static pressure was imposed on the inlet and outlet. However, when the blood flow was simulated in the aorta, the aortic stresses, luminal volume and radial dilation were respectively reduced by 6.4–7.5%, 3.0% and 1.1–2.9%. In fact, the continual blood flow was maintained by the pressure gradient along the aortic course. In other words, further along the aortic pathway, lower the luminal pressure became. This could explain why the stresses and aortic diameters in FSI simulation with flow were lower compared to control model and this tendency seemed to be more significant for the descending aorta (Table 1).

Considering the different results between simulations with or without blood flow, it was necessary to mimic the non-uniform hydrodynamic pressure ambient in the aorta as a consequence of the flowing blood. The wall stress resulting from blood pressure could be 0.48 MPa, while the wall shear stress (WSS) due to the blood flow was < 1.5 Pa at different aortic sections (see Additional file 3: Appendix S3). In other words, WSS was negligible in terms of its magnitude compared to wall stress. Although WSS could not lead to aortic dissection directly, its variable distributions have been suggested to induce aortic aneurysm progression and aortic tissue remodelling through a complex interplay between vascular cellular migration and extracellular matrix homeostasis [25–27]. Therefore, it was still essential to simulate the blood flow and its interaction with the aortic wall while studying the WSS effects on aortic pathologies and diseases.

### Relative contribution of aortic root motions to ascending aorta dissection

Both effects of AR axial and in-plane motion on aortic responses were evaluated by imposing downward and in-plane displacement on proximal AA end. Similar with other researches [2, 9], the peak aortic von Mises and circumferential stress were always located at the superior artery branches. The AR traction was previously postulated to increase proximal AA transection risk by elevating its longitudinal stress [2, 9]. Therefore, the stress levels were also evaluated by respectively averaging the von Mises, circumferential and longitudinal stresses of the AA section 2 cm above the AR under different loading conditions (Table 2). The AR axial motion contributed to 40% increase of AA longitudinal stress, in spite of the previously reported higher values 50–150% [2, 9]. However, the longitudinal motion elevated the AA von Mises and longitudinal stress by 37.6% and 32.6% in our study, which contradicted with its negligible influences on these stresses in [2, 9]. Another difference was the location of peak aortic longitudinal stress, which was always at the aortic arch interior curvature in our study but at the superior artery intersections previously [2, 9]. These discrepancies could be attributed to two reasons.

On one hand, both researches [2, 9] assumed aortic wall to be linear elastic material with a Young’s modulus of 3 MPa. Aorta is actually a complex fiber-reinforced composite structure displaying highly nonlinear responses. Previous aortic uniaxial stretch tests [28, 29] suggested the stiffness of young healthy samples continuously increase as the stretch ratio was higher than 1.20. With a luminal pressure of 120 mmHg, the peak aortic stretch ratio reached 1.36 previously in [28] and 1.48 in our study. Therefore, the aortic stiffness under the pressure of 120 mmHg with or without AR motion should not be defined as constant. Moreover, the elastic modulus of 3 MPa in these two studies [2, 9] might be stiffer compared to the dynamic stiffness of healthy aorta, which was found less than 1.5 MPa at the stress level of 74 kPa corresponding to a stretch ratio range of 1.18–1.49 [30]. Admittedly, the transversely isotropic material was a limitation of our study, but the behavior of healthy aortic wall was proved practically isotropic with the stretch ratio less than 1.8 [28, 29]. The transversely isotropic hyper-elastic material, the parameters of which were previously obtained by fitting aortic stretch curves [14, 17], was considered a good approximation to the aortic responses within the loading levels of our study (maximal stretch ratio < 1.50).

On the other hand, a toroidal coordinate system was constructed to convert the global stresses into local circumferential and longitudinal stress in both previous researches [2, 9]. However, this approach might be questionable since the complex geometry of the aorta was beyond the ability of a single global system to convert into local stresses. In this work, each element axis was oriented along the aortic longitudinal direction (see Additional file 3: Appendix S4) during the model discretization process. The circumferential or longitudinal stress could be converted according to each local element system in post-processing. In this way, the conversion of the circumferential and longitudinal stresses could avoid being affected by the aortic geometry.

The circumferential stress at AA in our work was always less than 0.20 MPa with the longitudinal component only half of its magnitudes (see Table 2). All the stresses in this study were found to be negligible compared with the yield stress (1.18 ± 0.12 MPa in circumferential and 1.21 ± 0.09 MPa in longitudinal directions) reported in [31] or the tensile rupture stress (1.27 MPa) of thoracic aorta published in [28]. Furthermore, the peak stretch ratio of AA was always less than 1.50 under all loading conditions and was also well below the previously recorded stretch failure of 2.1 [28]. Therefore, despite its effects of increasing AA longitudinal stress, the AR downward motion associated with heart traction could hardly induce aortic transverse dissection or add the injury risks to the healthy populations. The effects of AR downward motion remain to be investigated among other populations since our results were obtained with healthy subjects (normal aortic material, morphology and hemodynamics).

Compared with the downward motion, the AR in-plane displacement did not seem to alter the aortic stresses especially for the AA segment, which was still true even with the in-plane displacement magnitudes doubled. Although comparable with AR axial displacement (8.9 ± 1.8 mm), the AR in-plane motion (5.5 ± 1.7 mm) was inappreciable versus the distance (130 mm in our model) between the AR and brachiocephalic artery. Thus, the in-plane motion could barely change the aortic length (longitudinal deformation). Since aortic inflation was mainly the consequence of luminal pressure, the in-plane motion hardly induced circumferential deformation, either. Without longitudinal or circumferential deformation, the stress level would not be modified.

Although the AR axial or in-plane motion did not seem to elevate the aortic dissection risks in this study, additional mechanisms should account to aortic dissection. This injury should still be related to the factors increasing aortic wall stress and reducing aortic strength. The aortic stress could be enhanced by such factors as hypertension and aortic dilation. Cardiovascular diseases like aortic insufficiency would increase AR axial motion through ventricular compensation [2]. This increased motion could additionally elevate the aortic wall stress in subjects with higher aortic stiffness attributed to higher ages and vascular diseases (e.g. Marfan syndrome and atherosclerosis). Moreover, in these vascular diseases, the aortic strength would also be jeopardized with the aortic tissue remodelled. When the local aortic stress exceeds what the aortic tissue can resist, the aortic dissection might occur.

## Conclusions

The AR in-plane motion was analysed with the MRI data from 25 volunteers. The in-plane displacement increased during systole and regressed in diastole. The in-plane movement was found to be comparable to the axial motion, with its mean value (± standard deviation) 5.5 ± 1.7 mm. The X and Y components of in-plane motion were respectively 3.1 ± 0.9 mm, − 4.4 ± 1.7 mm under MRI reference system and 3.1 ± 1.5 mm, 3.0 ± 1.3 mm under PDA system. Blood flow should be simulated with FSI approach considering the lower values of aortic diameters, volumes and stresses as a result of hydrodynamics. The AR downward displacement did not improve AA’s vulnerability to dissection since the resulting 40% increase of longitudinal stress was still trivial against the aortic yield stress. With inducing negligible aortic circumferential or axial deformation, AR in-plane motion had no effect on aortic stress levels.

## Additional files

**Additional file 1: Appendix S1.** Mesh convergence analysis.

**Additional file 2: Appendix S2.** The equation of state (EOS) for blood.

**Additional file 3: Appendix S3.** Stress distribution of structural and FSI simulations.

**Additional file 4: Appendix S4.** Blood velocity profile and WSS at AA, PDA and DDA.

**Additional file 5: Appendix S5.** Element axis orientation.
