# Supplementary material for: Investigating heartbeat-related in-plane motion and stress levels induced at the aortic root
Source: Biomed Eng Online. 2019 Feb 26;18:19. doi: 10.1186/s12938-019-0632-7 (PMC6391796; doi:10.1186/s12938-019-0632-7)
Supplement: Supplementary file 1 — Additional file 1: Appendix S1. Mesh convergence analysis. [file 12938_2019_632_MOESM1_ESM.pdf]

Appendix S1. Mesh convergence analysis

The von Mises stress distribution is similar among the three models of different element sizes. The maximum stress always occurred at the same region (the intersection of the superior arteries) but the stress magnitudes differed among the three models. As displayed in Table A, the difference of peak aortic stress was less than 5% between the models of 3.0e+4 and 1.0e+5 elements while the difference was below 1.0% between the models of 1.0e+5 and 3.0e+5 elements. Accordingly, the aortic model of 1.0e+5 elements was chosen for the following simulations.

Table A.1 Peak von Mises values occurred in the three models during mesh convergence analysis

Element number	3.0e+4	1.0e+5	3.0e+5
Node number	4.5e+4	1.3e+5	3.8e+5
Peak stress (MPa)	0.019	0.020	0.020
