# Supplementary material for: Investigating heartbeat-related in-plane motion and stress levels induced at the aortic root
Source: Biomed Eng Online. 2019 Feb 26;18:19. doi: 10.1186/s12938-019-0632-7 (PMC6391796; doi:10.1186/s12938-019-0632-7)
Supplement: Supplementary file 2 — Additional file 2: Appendix S2. The equation of state (EOS) for blood. [file 12938_2019_632_MOESM2_ESM.pdf]

Appendix S2. The equation of state (EOS) for blood

The EOS is a thermodynamic equation which provides a mathematical relationship between two or more state variables (for example: temperature, pressure) associated with a material. The EOS is often used to correlate densities of materials to temperatures and pressures especially in the situations when the material is subjected to very high strain rates and when the material pressures are much higher than yield stress. In this study, the linear Gruneisen EOS was used to describe the relation between blood pressure and density, which could be computed with the simplified equation (B.1):

$$p = (\rho - \rho_0) \cdot C^2 \qquad \text{(B.1)}$$

where p is the blood pressure; ρ and ρ_0 correspond to the current and initial blood densities; C is the speed of sound propagating through the blood. C could also be calculated by the Newton-Laplace equation (B.2):

$$C = \sqrt{\frac{K}{\rho}} \qquad \text{(B.2)}$$

where K is the blood bulk modulus and ρ was assumed to be equal to initial density for simplifying the sound speed calculation. The sound speed passing through blood was determined to be 1543.0m/s.

Since it is impossible to prescribe pressure to Eulerian meshes directly in LS-DYNA, the blood relative volume was computed according to the equation (B.3):

$$v_r = \frac{v}{v_0} = \frac{\rho_0}{\rho} = \frac{\rho_0 \cdot C^2}{p + \rho_0 \cdot C^2} \qquad \text{(B.3)}$$

where v_0, v and v_r correspond to initial, current and relative blood volume.
