# Supplementary material for: Investigating heartbeat-related in-plane motion and stress levels induced at the aortic root
Source: Biomed Eng Online. 2019 Feb 26;18:19. doi: 10.1186/s12938-019-0632-7 (PMC6391796; doi:10.1186/s12938-019-0632-7)
Supplement: Supplementary file 3 — Additional file 3: Appendix S3. Stress distribution of structural and FSI simulations. [file 12938_2019_632_MOESM3_ESM.pdf]

Appendix S3. Stress distribution of structural and FSI simulations

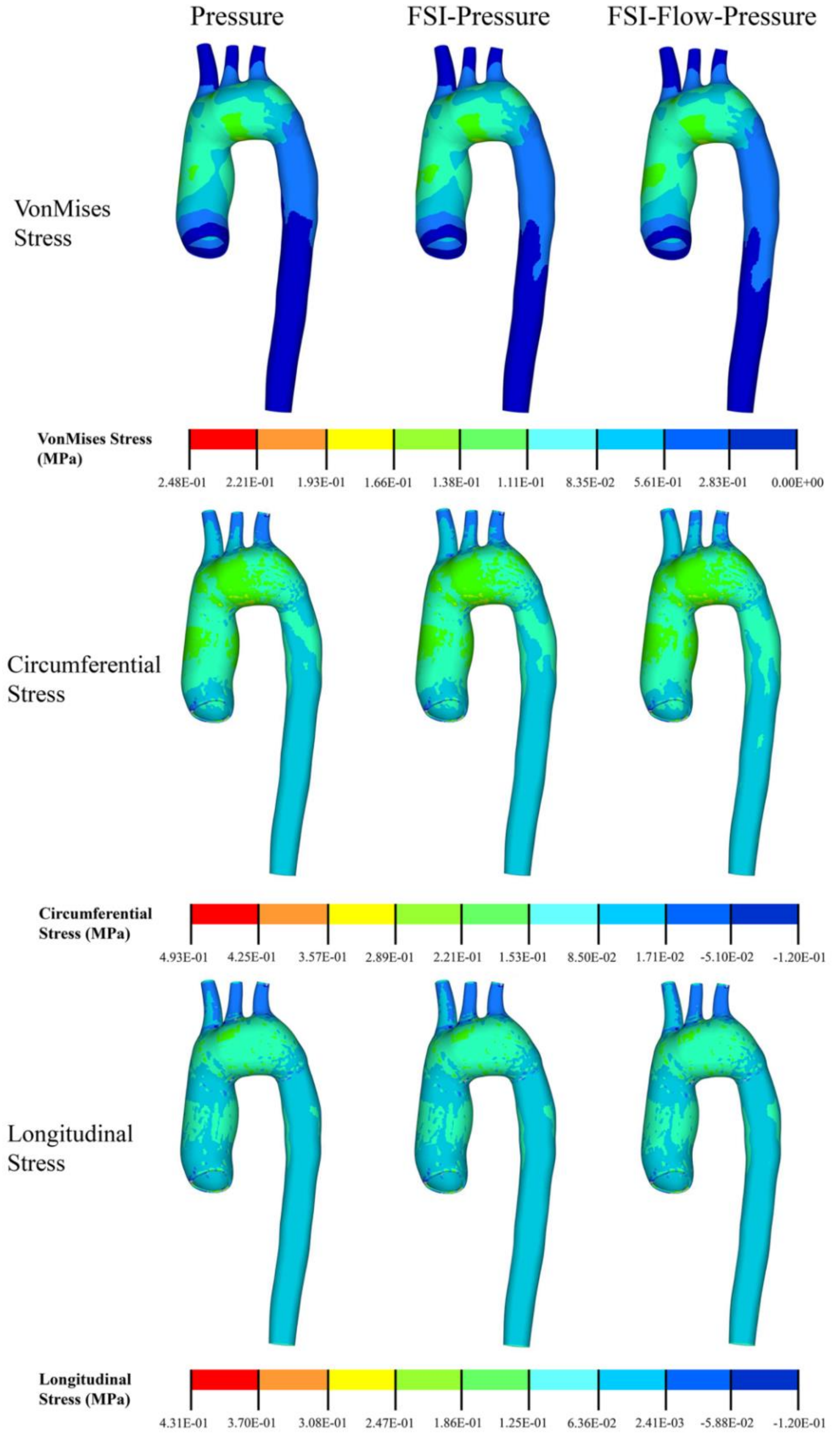

Fig. C. 1 VonMises, circumferential and longitudinal stress distribution of the control model and FSI simulations
