# Supplementary material for: Investigating heartbeat-related in-plane motion and stress levels induced at the aortic root
Source: Biomed Eng Online. 2019 Feb 26;18:19. doi: 10.1186/s12938-019-0632-7 (PMC6391796; doi:10.1186/s12938-019-0632-7)
Supplement: Supplementary file 4 — Additional file 4: Appendix S4. Blood velocity profile and WSS at AA, PDA and DDA. [file 12938_2019_632_MOESM4_ESM.pdf]

Appendix S4. Blood velocity profile and WSS at AA, PDA and DDA

The blood velocity of each node at different aortic sections (AA, PDA and DDA) was exported from the simulation. With the fitting tools of MATLAB R2013a (The Mathworks, Natick, MA, USA), the cubic spline interpolation was used to fit the blood flow profile at each section (Fig. D. 1). WSS corresponds to the friction force from the flowing blood at the vessel wall and depends on the spatial velocity gradient at the wall. It can be computed with the simplified equation (D.1):

$$\mathrm{WSS} = \mu \left(\frac{\delta u}{\delta x}\right)_{x=0} \qquad \text{(D.1)}$$

where μ is the blood dynamic viscosity, u the blood flow velocity along the lumen direction and x the distance from the wall along its inward normal direction. In Fig. D. 2 was displayed the WSS distribution at AA, PDA and DDA.

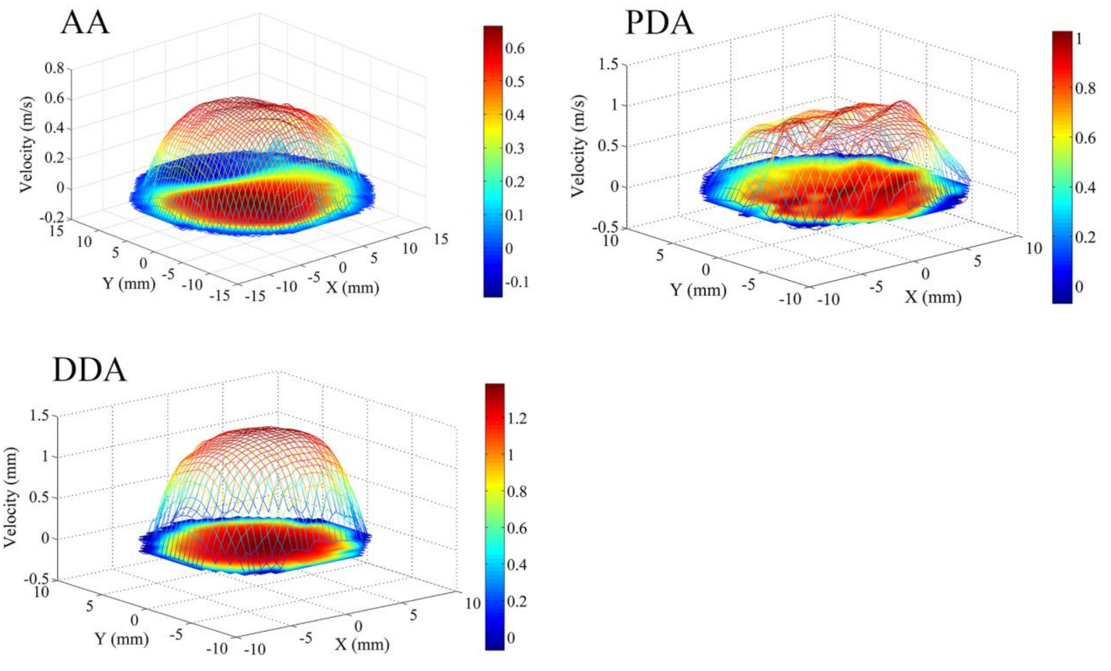

Fig. D. 1 Blood velocity profile at AA, PDA and DDA

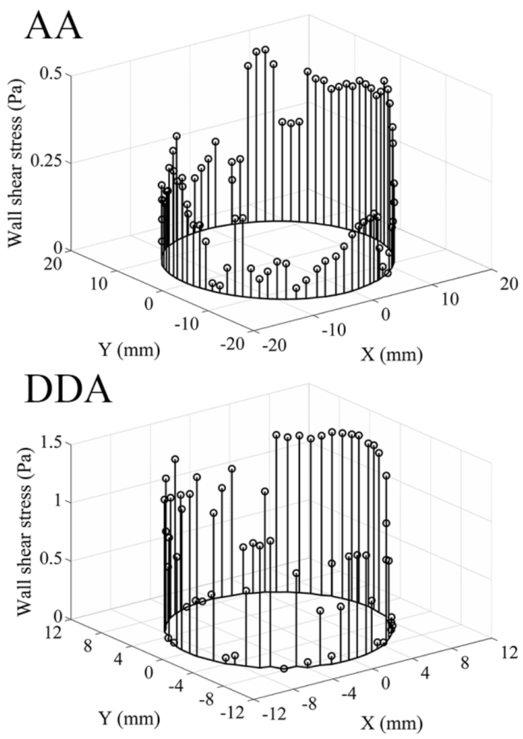

Fig. D. 2 Wall shear stress distribution at AA, PDA and DDA
