# Supplementary material for: Investigating heartbeat-related in-plane motion and stress levels induced at the aortic root
Source: Biomed Eng Online. 2019 Feb 26;18:19. doi: 10.1186/s12938-019-0632-7 (PMC6391796; doi:10.1186/s12938-019-0632-7)
Supplement: Supplementary file 5 — Additional file 5: Appendix S5. Element axis orientation. [file 12938_2019_632_MOESM5_ESM.pdf]

Appendix S5. Element axis orientation

In order to obtain the circumferential and longitudinal stress of the aortic wall, the element axis was firstly oriented during mesh construction process. In LS-Prepost, the ‘direction’ function in ‘element editing’ submenu was used to orient the axis of the selected aortic shell elements along lumen longitudinal direction (Fig. E. 1). The aortic shell elements were subsequently offset with an uniform 2mm thickness to generate the brick elements, the axis of which would also be consistently oriented. During post-processing, the aortic wall stress could be displayed according to element coordinate system, where the XX (Fig. E. 2) component corresponded to the circumferential stress and YY component (Fig. E. 3) to the longitudinal stress.

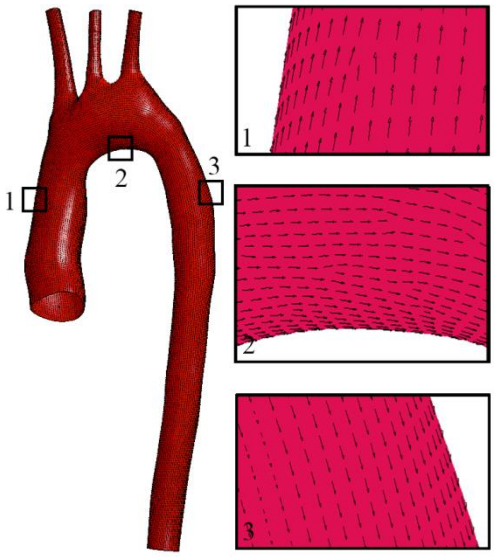

Fig. E. 1 Element axis of 2D aortic shell elements

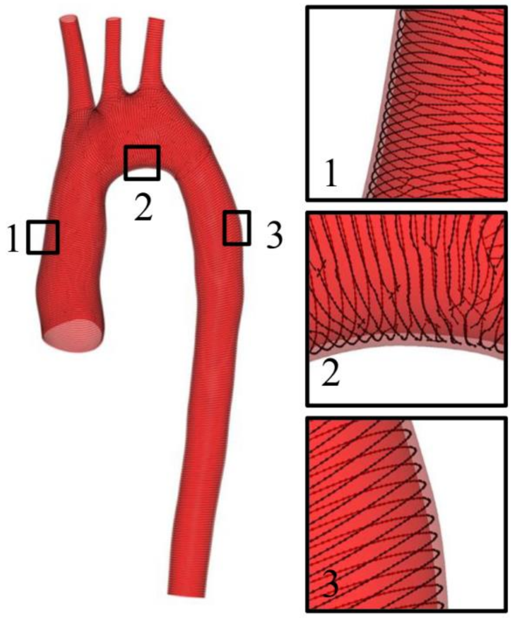

Fig. E. 2 Circumferential stress distribution on 3D aortic wall

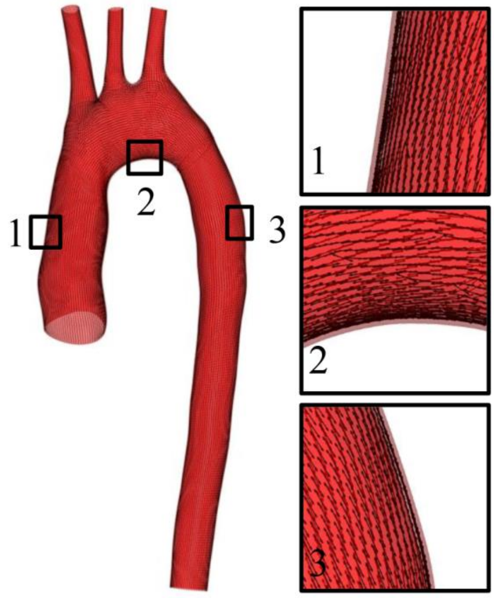

Fig. E. 3 Longitudinal stress distribution on 3D aortic wall
